# Nutrient density and the collaborative impact of exogenous enzyme blend on the performance of broiler chicken

**DOI:** 10.5713/ab.24.0233

**Published:** 2024-10-24

**Authors:** Jun Young Mun, Habeeb Tajudeen, Sang Hun Ha, Jun Hyung Lee, Anushka Lokhande, Santosh Laxman Ingale, Jin Soo Kim, Min Ju Kim

**Affiliations:** 1 Department of Animal Industry Convergence, Kangwon National University, Chuncheon, 24341, Korea; 2 Department of Animal Biosciences (ABSc), University of Guelph, Guelph, ON N1G 2W1, Canada; 3 Advanced Enzyme Technologies Ltd., Thane 400604, India; 4 Institute of Applied Humanimal Science, Hankyong National University, Ansung 17579, Korea; 5 School of Animal Life Convergence Science, Hankyong National University, Ansung 17579, Korea

**Keywords:** Bird Performance, Digestibility, Microbiota, Nutrient Deficiency, Poultry

## Abstract

**Objective:**

This study evaluates the collaborative effect of exogenous enzyme blend and dietary nutrient density on the performance of broiler chicken.

**Methods:**

A total of 600 Ross 308 broiler chickens with same average initial body weight were randomly assigned to 5 treatments. Each treatment contained 8 replicates, and 15 birds per replicate. The diets included a control (CON) starter/finisher (S/F) diet with metabolizable energy (ME) 3,100/3,200 in Kcal/kg and crude protein (CP) content 22.0.0/20.00 in % as (S/F 3,100/3,200 Kcal/kg + CP [22.00/20.00]%), S/F with ME 3,060/3,150 Kcal/kg + CP (21.50/19.50)% with and without the exogenous enzyme blend as (S/F 3,060/3,150 Kcal/kg + [21.50/19.50]% with, and without the exogenous enzyme blend), and lastly, S/F with ME 3,010/3,100 Kcal/kg + CP (21.50/19.50)% with, and without the exogenous enzyme blend as (S/F 3,010/3,100 Kcal/kg + [21.50/19.50]% with, and without the exogenous enzyme blend). The impact of the treatments was tested on growth performance, nutrient digestibility, blood metabolites, intestinal microflora, and morphology of broiler chicken.

**Results:**

The inclusion of exogenous enzyme blend in the nutrient-deficient diet S/F 3,060/3,150 + 21.50/19.50 increased (p<0.05) broilers body weight, feed conversion ratio, nutrient digestibility of CP, gross energy, phosphorus, and blood phosphorus, with tendency (p<0.10) of higher dry matter. The treatment also showed lower (p<0.05) total anaerobic bacteria, *coliform*, and higher (p<0.05) villus height (VH) in the jejunum, with tendencies (p<0.10) of higher *lactobacillus* in the ileum and caecum, and higher tendency (p<0.10) of VH in duodenum and ileum.

**Conclusion:**

We concluded that the improved performance could be attributed to the potency of S/F 3,060/3,150 + 21.50/19.50 supplemented with 0.05% of the multienzyme to reduce the level of potential pathogenic bacteria with an increased level of positive bacteria, which in turn creates an enabling intestinal villi structure in broiler chicken.

## INTRODUCTION

The dearth of feed ingredients and increasing environmental contamination in poultry production have gathered global attention. Diets containing low nutrients supplemented with enzymes may be considered an option. The incorporation of exogenous enzymes such as amylase, mannanase, phytase, protease, and xylanase has the ability to maintain, and improve the performance of broiler chicken fed low-nutrient diets consisting of corn-wheat-soybean meal (SBM) [1], a typical ingredient utilized for broiler feed formulation [2]. These ingredients are traditionally valued on account of their availability and nutritional balance. However, a range of anti-nutritional components linked to their fibrous nature—including polymeric carbohydrates termed non-starch polysaccharides (NSPs) are included in feed items originating from plants [3]. The majority of NSPs in corn, SBM, and wheat include insoluble arabinoxylan and β-galactomannan, α-galactosides, and β-galactomannan, and arabinoxylan respectively [4].

Poultry including broiler chickens lack the adequate number of endogenous enzymes required for splitting the bonds of intricate polysaccharides found in feed ingredients originating from plants [5]. These polymers decrease the nutritional value of feeds by preventing endogenous enzymes from accessing the confined protein and starch in the cell wall [6]. The existence of NSP in broiler diets contributes to elevated intestinal viscosity, decreased nutrient digestibility, and altered gut physiology, leading to poor performance [3]. Exogenous multi-enzymes incorporated into corn-SBM and wheat-SBM-based diets reduced the effects of differing raw material quality, improved homogeneity and enhanced nutrient digestibility compared to single enzymes [7,8], by reducing the chemical composition of NSP passing through the ileum [9]. Despite the notion that multi-enzymes could enhance digestibility, their impact tends to be more obvious in nutrient-deficient diets [4].

Thus, the purpose of this experiment was to evaluate the impact of dietary nutrient density and the collaborative effect of exogenous enzyme blend on the growth performance, nutrient digestibility, blood metabolites, intestinal microflora and morphology in broilers fed corn-wheat-SBM diets.

## MATERIALS AND METHODS

### Animal care and ethical statement

This experiment was conducted at the facility of Kangwon National University after receiving approval from the Institutional Animal Care and Use Committee with ethical code KW-220413-1.

### Animal, diets, and experimental design

A total of 600 broilers (Ross 308) with same average initial body weight (BW) were randomly assigned to 5 treatments. Each treatment contained 8 replicates, and 15 birds per replicate. The diets included a control (CON) starter/finisher (S/F) diet with metabolizable energy (ME) 3,100/3,200 in Kcal/kg, and crude protein (CP) content 22.0.0/20.00 in % as (S/F 3,100/3,200 Kcal/kg + CP, [22.00/20.00]%), S/F with ME 3,060/3,150 Kcal/kg + CP (21.50/19.50)% with and without the exogenous enzyme blend as (S/F 3,060/3,150 Kcal/kg + [21.50/19.50]% with, and without the exogenous enzyme blend), and lastly, S/F with ME 3,010/3,100 Kcal/kg + CP (21.50/19.50)% with, and without the exogenous enzyme blend as (S/F 3,010/3,100 Kcal/kg + [21.50/19.50]% with, and without the exogenous enzyme blend). The experimental diets were fed in a mash form for 35 days in 2 phases (d 1 to 21, phase I; and d 22 to 35, phase II). All diets met or exceeded the nutrient requirements recommended by the Ross 308 (Aviagen, 2019) management guide [10] (Tables 1, 2). The birds were raised on floor enclosures covered with rice hulls. A self-feeder and a hanging bell drinker were installed in each pen to enable unrestricted access to food and water. The room was heated to 34°C for the first 5 d and was progressively lowered according to normal management procedures until a temperature of 23°C was achieved, with lighting allowed for 23 h/d.

### Enzyme preparation

The exogenous enzyme blend used in the present study known as DigeGrain Delta was provided by Advanced Enzyme Technologies Ltd. (Thane, India). The multienzyme is a mix of mannanase, protease, amylase, phytase, and xylanase synthesised by controlled fermentation of *Bacillus amyloliquefaciens*, *Bacillus licheniformis*, *Trichoderma citrinoviride*, and *Pichia pastoris*, respectively. The pure enzyme product provides 2,500,000 mannanase units (MNU)/kg of β-mannanase, 800,000 bacterial amylase units (BAU)/kg of amylase, 1,000,000 phytase temperature units (FTU)/kg of phytase, 1,000,000 protease catalytic units (PCU)/kg of protease, 600,000 xylanase units (XU)/kg of xylanase, and 1 unit of amylase is the amount of enzyme liberating 1 μmoL of glucosidic linkage from a dextrinized starch substrate per minute at 6.6 pH and 30°C. A unit of protease is the quantity of enzyme liberating 1 μg of phenolic compound (tyrosine equivalent) from casein substrate per minute at 7.0 pH and 37°C, 1 unit of β-mannanase is the quantity of enzyme, which liberates 1 μmoL of total reducing sugar (glucose equivalent) from mannan substrate (Locust bean gum) per minute at 5.3 pH and 50°C. A unit of xylanase is defined as the quantity of enzyme liberating 1 μmoL of reducing sugar (xylose equivalent) from xylan substrate (Beechwood xylan) per minute at 5.3 pH and 50°C, and 1 unit of phytase is defined as the amount of enzyme, which liberates 1 μmoL of inorganic phosphate from sodium phytate substrate per minute at 5.0 pH and 50°C.

### Data and sample collection

The birds were weighed separately on d 21 and d 35 of the experiment, and at the conclusion of the experiment, un-consumed feed was weighed, and feed intake (FI) was determined. Body weight gain and FI were measured from each replicate, and feed conversion ratio (FCR) were corrected for the weight of dead birds. Nutrient balance trials were conducted at the end of the feeding trial to determine the apparent ileal and total digestibility of dry matter (DM), CP, gross energy (GE), calcium (Ca), and phosphorus (P). Two birds from each replicate were allocated in individual cages (one bird/cage) to facilitate the collection of excreta samples. The diets containing 0.25% chromium oxide as an indigestible marker were given, and excreta and ileal samples (about 100 g/d per bird) were collected from each bird. For the Ileal sample collection, a total of 80 birds (2 birds in each replicate from the treatment) were euthanized and the ileal digesta was collected from the distal part. The samples were dried in a forced air-drying oven at 60°C for 72 h and ground in a Wiley laboratory mill (Thomas Model 4 Wiley Mill; Thomas Scientific, Swedesboro, NJ, USA) using a 1-mm screen. The total nutrient digestibility was calculated as:


Total nutrient digestibility (%)=100-[100×(% Cr in feed/% Cr in excreta)×(% nutrient in excreta)/% nutrient in feed)].

The apparent ileal digestibility (AID) was calculated as:


Nutrient AID (%)=100-[100×(% Cr in feed/% Cr in ileum)×(% nutrient in ileum/% nutrient in feed)].

On d 21 and d 35, a 10-mL blood sample was collected by jugular vein puncture from 2 randomly selected broilers in each pen using a disposable vacutainer tube containing sodium heparin as an anticoagulant (Becton Dickinson, Franklin, NJ, USA). After centrifugation (3,000×g for 15 min at 4°C), serum samples were separated and stored at −20°C and later analyzed for concentrations of cholesterol, triglyceride, glucose, Ca, and P [11].

### Small intestinal morphology

On the last day of the experiment, a total of 80 birds (2 birds in each replicate from the treatment) were euthanized and intestinal samples were collected from the small intestine. Three cross-sections for each intestinal sample were prepared after staining with azure A and eosin using standard paraffin embedding procedures [12]. A total of 10 intact, well-oriented crypt-villus units were selected in triplicate for each intestinal cross-section from the duodenum, jejunum, and ileum. Villus height (VH) was measured from the tip of the villi to the villus crypt junction, and crypt depth (CD) was defined as the depth of the invagination between adjacent villi and villus width was measured at the mid of the villus. All morphological measurements (VH or CD) were made in 10-μm increments by using an image processing and analysis system (Optimus software version 6.5; Media Cybergenetics, North Reading, MA, USA).

### Chemical analysis

Experimental diets, excreta and ileal samples were analyzed in triplicate for DM, method 930.15; AOAC [13], CP method 990.03; AOAC [13], and ash method 942.05; AOAC [13]. The Ca, and P (Method 985.01; AOAC [13]). The GE were measured by a bomb calorimeter (Model 1261; Parr Instrument Co., Moline, IL, USA), and chromium concentration was determined with an automated spectrophotometer (Jasco V-650; Jasco Corp., Tokyo, Japan) according to the procedure of Fenton and Fenton [14]. Commercial kits (Fujifilm Corp., Saitama, Japan) were used for the analysis of serum metabolites using an automated chemical analyser (Fuji Dri-chem 3500i; Fujifilm Corp., Japan).

### Microbial analysis

At the end of this trial, the microbiological assay of intestinal digesta and faecal samples was carried out using the procedure suggested by Choi et al [15]. In detailed; Ileal and rectal samples were collected from previously euthanized 80 birds (2 birds in each replicate from the treatment), and 1 g of mixed content was diluted with 9 mL of Butterfield’s phosphate buffer solution, followed by further serial dilutions in Butterfield’s phosphate buffer dilution solution. Duplicate plates were then inoculated with 0.1 mL sample and incubated. The microbial groups enumerated were the total anaerobic bacteria (Tryptic soya agar), *Lactobacillus* spp. (MRS agar + 0.02% NaNO3 + 0.05% L-cysteine hydrochloride monohydrate), *Clostridium* spp. (Tryptose sulphite cycloserine agar, Oxoid, Hampshire, UK) and coliforms (violet red bile agar; Difco Laboratories, Detroit, MI, USA). The anaerobic conditions during the assay of *Clostridium* spp. were created by using a gas-pak anaerobic system (BBL, No. 260678; Difco, Detroit, MI, USA). The microbial populations were log-transformed before statistical analysis.

### Statistical analysis

Data generated in the present study were subjected to statistical analysis using the general linear model procedure of SAS (SAS Inst. Inc., Cary, NC, USA) in a randomized complete block design. When significant differences were identified among treatment means, they were separated using Tukey’s honestly significant difference test. The pen was used as the experimental unit for the analysis of growth performance, digestibility coefficient, blood metabolites, faecal microflora and intestinal morphology. Probability values of p<0.05 were considered significant, and p<0.10 as tendency.

## RESULTS

### Bird performance

The impact of dietary nutrient density and supplementation of exogenous enzyme blend on the growth performance of broiler is shown in Table 3. In the starter phase, broilers fed S/F 3,060/3,150 + 21.50/19.50 supplemented with exogenous enzyme blend had higher (p<0.05) BW compared with S/F 3,060/3,150 + 21.50/19.50 without enzyme supplementation, and S/F 3,010/3,100 + 21.50/19.50 without enzyme, while FCR was lower (p<0.05) in the exogenous enzyme blend supplemented diets compared with S/F 3,010/3,100 + 21.50/19.50 without enzyme supplementation. In the finisher phase, BW was higher (p<0.05) in the exogenous enzyme blend supplemented diets compared with S/F 3,010/3,100 + 21.50/19.50 without the exogenous enzyme blend, while FCR was lower (p<0.05) in the enzyme blend supplemented diets compared with S/F 3,010/3,100 + 21.50/19.50 without the enzyme supplementation. In the overall days, the BW was higher (p<0.05) in S/F 3,060/3,150 + 21.50/19.50 supplemented with the enzyme blend at 0.05% compared with S/F 3,060/3,150 + 21.50/19.50 without the enzyme, and 3,010/3,100 + 21.50/19.50 without the exogenous enzyme blend. The FCR was lower (p<0.05) in the enzyme-supplemented diets compared with S/F 3,060/3,150 + 21.50/19.50 and S/F 3,010/3,100 + 21.50/19.50 without the enzyme blend. There was no significant difference in FI across all phases of the experimental treatments.

### Digestibility of nutrients

The impact of dietary nutrient density and supplementation of exogenous enzyme blend on the faecal and ileal digestibility (%) in broiler at d 35 is shown in Table 4. In the faeces, digestibility of CP was higher (p<0.05) in S/F 3,060/3,150 + 21.50/19.50 supplemented with the exogenous enzyme blend with tendencies (p<0.10) of higher DM, GE, and P digestibility compared with S/F 3,060/3,150 + 21.50/19.50 without the enzyme, and S/F 3,010/3,100 + 21.50/19.50 without the exogenous enzyme blend supplementation. There was no significant difference in the faecal Ca digestibility. The ileal DM showed higher tendency (p<0.10) in the S/F 3,060/3,150 + 21.50/19.50, and the 3,010.60/3,100 + 21.50/19.50 exogenous blend supplemented diet compared with S/F 3,010/3,100 + 21.50/19.50 without the enzyme blend supplementation. The ileal GE was higher (p<0.05) in S/F 3,060/3,150 + 21.50/19.50 supplemented with the exogenous enzyme blend compared with S/F 3,010/3,100 + 21.50/19.50 without the exogenous enzyme. The ileal CP digestibility showed higher tendency (p<0.10) in S/F 3,060/3,150 + 21.50/19.50 supplemented with the exogenous enzyme blend. The ileal P digestibility was higher (p<0.05) in S/F 3,060/3,150 + 21.50/19.50 supplemented with the exogenous enzyme blend compared with S/F 3,060/3,150 + 21.50/19.50 without the enzyme, and S/F 3,010/3,100 + 21.50/19.50 without the enzyme blend. There was no significant difference in the ileal Ca digestibility across all treatments.

### Blood metabolites

The impact of dietary nutrient density and supplementation of exogenous enzyme blend on blood metabolites in broiler is shown in Table 5. The blood glucose, triglyceride, cholesterol, and Ca showed no significant difference at d 21 and d 35. However, the blood P was higher (p<0.05) in S/F 3,060/3,150 + 21.50/19.50 supplemented with the exogenous enzyme blend at d 21 and d 35 compared with S/F 3,010/3,100 + 21.50/19.50 without the exogenous enzyme blend.

### Intestinal microflora

The impact of dietary nutrient density and supplementation of exogenous enzyme blend on the intestinal microflora of broiler at d 35 is shown in Table 6. The total anaerobic bacteria in ileum were lower (p<0.05) in S/F 3,060/3,150 + 21.50/19.50 supplemented with the exogenous enzyme blend compared with S/F 3,060/3,150 + 21.50/19.50, and 3,010/3,100 + 21.50/19.50 without the enzyme blend. There was no significant difference in the total anaerobic bacteria in the caecum across all treatments. *Lactobacillus* spp. showed higher tendency (p<0.10) in the ileum and caecum in S/F 3,060/3,150 + 21.50/19.50 supplemented with the exogenous enzyme. The *coliform* bacteria showed no significant difference in the ileum. However, *coliform* bacteria in the caecum were lower (p<0.05) in S/F 3,060/3,150 + 21.50/19.50 supplemented with the exogenous enzyme blend compared with S/F 3,060/3,150 + 21.50/19.50, and S/F 3,010/3,100 + 21.50/19.50 without the enzyme blend. There was no significant difference in *Clostridium* spp. in the ileum and caecum in all treatments.

### Intestinal morphology

The impact of dietary nutrient density and supplementation of exogenous enzyme blend on the intestinal morphology of broiler is shown in Table 7. In the duodenum, there was a higher tendency (p<0.10) in VH in the S/F 3,060/3,150 + 21.50/19.50 supplemented with the exogenous enzyme, however, there was no significant difference in CD, and VH/CD. In the jejunum, VH was higher (p<0.05) in S/F 3,060/3,150 + 21.50/19.50 supplemented with the exogenous enzyme blend compared with S/F 3,010/3,100 + 21.50/19.50 without the enzyme blend. There was no significant difference in CD and VH/CD in all treatments. In the ileum, a tendency (p<0.10) was observed in VH in the S/F 3,060/3,150 + 21.50/19.50 supplemented with the exogenous enzyme, however, there was no significant difference in the CD, and VH/CD across all treatments.

## DISCUSSION

In this experiment, a decrease in BW and an increase in FCR was observed when dietary ME and CP were reduced at the starter, finisher, and overall phases. This result agrees with the study of Mirshekar et al [16]; Dean et al [17] where broilers fed diets on low CP and ME had poor BW and FCR. Although the FI was not significantly impacted by the reduced CP and ME, a numerical increase was observed. This is based on the established inverse relationship between FI and dietary nutrient levels. Birds fed on nutrient-deficient diets often ingest more feed to meet their nutrient and energy requirements.

The supplementation of the enzyme blends to the diet with reduced ME and CP enabled growth performance comparable to the CON diet containing non-reduced ME and CP. The theories of science listed below can be used to explain this: The supplementation of multienzyme improved FCR without an effect on FI. This can be interpreted as the improvement resulted from better nutrient utilization [18]. The inability of broiler chickens to effectively digest NSP due to the limited quantity of endogenous enzymes such as β-mannanase, α-galactosidase, and arabinoxylanase in the gut typically results to the utilization of more energy by broilers. Exogenous enzyme blend performs in corn-wheat-SBM diets primarily by disintegrating the plant cell wall components encapsulating nutrients in low-viscosity diets. Hence, assisting in converting the NSP into short-chain carbohydrates available for broiler utilization [2]. The enzyme employed in this study contains amylase, protease, mannanase, and xylanase which may improve the digestibility of energy and protein [19]. Xylanase in particular assists in degrading xylan in the distal region of the digestive tract leading to the formation of oligosaccharide prebiotics [20]. Thus, the supplementation of an exogenous enzyme blend gives room for lower CP and ME in diets without compromising the performance of broilers as shown in the current study.

The supplementation of enzyme blend to lower ME and CP diets not only increased the digestibility of faecal DM, GE, and CP but also increased ileal DM, GE, P, and enhanced blood P levels. This improvement may be affiliated with the blend of amylase, protease, mannanase, and xylanase capable of disintegrating dietary NSP as earlier explained. In agreement, Lu et al [21] stated that the combined supplementation of glucanase, phytase, and xylanase enhanced the CP, DM, GE, and P in broilers fed corn-SBM-diets deficient in ME, CP, Ca, and P.

Modifying the microbiota quality by enhancing the level of eubiotic bacteria is proportional to a reduction in the number of pathogenic bacteria in the gut, and this may be achieved via nutrient-deficiency practice supplemented with an enzyme blend [22]. Our study shows that the level of potential pathogenic bacteria such as total anaerobic bacteria in the ileum, and coliform bacteria in the caecum was lower in the nutrient-deficient diet supplemented with the exogenous enzyme blend. This may be due to the minute quantity of nutrients accessible for fermentation by pathogenic bacteria [18]. It is noteworthy to mention that one of the most important variables influencing the immune and physiological systems of broiler chickens is their gut microbiome. Pathogenic bacteria such as coliforms and total anaerobic bacteria intensify illnesses and reduce hens’ ability to thrive [23]. Although there was no significant difference in the level of beneficial bacteria such as Lactobacillus in this study, a tendency for higher levels of ileal and caecal *Lactobacillus* spp. was noticed in the nutrient-deficient diets supplemented with an exogenous enzyme blend. It has been previously explained that xylanase has the ability to degrade NSP such as arabinoxylan found in plant-based feed ingredients to synthesise short-chain fatty acids (SCFA) in the distal region of the broiler gut [24]. We proposed the derived SCFA may have created a conducive atmosphere to lactic acids such as *Lactobacillus* spp. as shown in the nutrient-deficient diet supplemented with an exogenous enzyme blend. The prevalence of lactic acid hinders the spread of harmful bacteria such as coliform and total anaerobic bacteria as shown in this study which shares similarities with a report by Yaghobfar and Kalantar [25]. In their study, the addition of β-glucanase and phytase resulted in a higher level of Lactobacillus and a decline in the *Escherichia coli* population in the ileum. Phytase is popularly known for decreasing diet buffer tendency by altering the digesta pH and microbial quantity in the ileum.

Intestinal architecture such as CD, VH, and their ratios are an important indicators of nutrient digested. A higher villus and a smaller crypt are signs of improved digested nutrients coupled with reduced mucosal tissue regeneration [26]. The current study showed higher VH in the jejunum and a tendency towards a higher VH in the duodenum and ileum in the nutrient-deficient diet supplemented with an exogenous enzyme blend. The treatment also showed numerically lower CD in the duodenum, jejunum, and ileum. The corn-SBM and wheat employed as the main ingredient source in this experiment contain β-mannan and arabinoxylans respectively. These polysaccharides augment the digesta viscosity that triggers a change in the intestinal structure leading to a shorter VH and longer CD as shown in our treatments without the enzyme supplementation [25]. We speculate that the higher VH and lower CD in the enzyme-supplemented diets was due to its mannanase and xylanase components inducing the formation of manno- and xylo-oligosaccharides in the gut of broilers. Similar reports by Ravn et al [27]; Roofchaei et al [28] observed an increase in villus length in diets supplemented with a combination of β-glucanase, phytase, and xylanase enzyme.

## CONCLUSION

In conclusion, broilers fed nutrient-deficient S/F 3,060/3,150 + 21.50/19.50 corn-wheat-SBM diets incorporated with exogenous enzyme blend at level 0.05% showed higher growth performance and digestibility of nutrients. This may have resulted from the potency of the treatment to reduce the level of potential pathogenic bacteria with an increased level of beneficial bacteria, which in turn creates an enabling intestinal villi structure.

## Figures and Tables

**Table 1:** Ingredient and chemical composition of basal diet, as-fed basis (Phase I)

Items	Value				
Metabolizable energy (Kcal/kg)	3,100	3,060	3,060	3,010	3.010
Crude protein (%)	22.00	21.50	21.50	21.50	21.50
Enzyme (%)^1)^	0	0	0.05	0	0.05
Ingredients (%)					
Corn	49.27	54.63	54.58	55.79	55.74
Wheat	5.00	5.00	5.00	5.00	5.00
Soybean meal 45%	32.36	27.98	27.98	27.97	27.97
Fish meal	2.00	2.00	2.00	2.00	2.00
Soy-oil	4.93	2.82	2.82	1.84	1.84
Threonine 98.5%	0.00	0.03	0.03	0.03	0.02
Lysine 78%	0.03	0.13	0.13	0.12	0.12
Methionine 50%	0.52	0.48	0.48	0.47	0.47
Choline	0.10	0.10	0.10	0.10	0.10
Limestone	1.65	1.49	1.49	1.49	1.49
MDCP	1.29	1.09	1.09	1.09	1.09
Salt	0.20	0.20	0.20	0.20	0.20
Mineral premix^2)^	0.20	0.20	0.20	0.20	0.20
Vitamin premix^3)^	0.30	0.30	0.30	0.30	0.30
Corn gluten	2.15	3.55	3.55	3.41	3.41
Enzyme	-	-	0.05	-	0.05
Total	100.00	100.00	100.0	100.0	100.0
Chemical composition, calculated (%)					
Metabolizable energy (Kcal/kg)	3,100	3,060	3,060	3,010	3,010
Crude protein	22.00	21.50	21.50	21.50	21.50
Calcium	1.00	0.90	0.90	0.90	0.90
Available phosphorus	0.45	0.40	0.40	0.40	0.40
SID Lysine	1.20	1.17	1.17	1.17	1.17
SID TSAA	0.94	0.90	0.90	0.90	0.90
Threonine	0.80	0.77	0.77	0.77	0.77
Tryptophan	0.25	0.22	0.22	0.22	0.22

MDCP, monodicalcium phosphate; SID, standardized ileal digestibiliy; TSAA, total sulfur amino acids; MNU, mannanase units; BAU, bacterial amylase units; FTU, phytase temperature units; PCU, protease catalytic units; XU, xylanase units.

1) The multienzyme is a mix of mannanase, protease, amylase, phytase, and xylanase synthesised by controlled fermentation of *Bacillus amyloliquefaciens*, *Bacillus licheniformis*, *Trichoderma citrinoviride*, and *Pichia pastoris* respectively. The pure enzyme product provides 2,500,000 MNU/kg of β-mannanase, 800,000 BAU/kg of amylase, 1,000,000 FTU/kg of phytase, 1,000,000 PCU/kg of protease, 600,000 XU/kg of xylanase.

2) Supplied per kg diet: 10,000 IU Vit A (retinyl acetate), 4,500 IU Vit D_3_ (cholecalciferol), 65 mg Vit E (DL-α-tocopheryl acetate), 1.5 mg Vit B_1_ (thiamine), 12 mg Vit B_2_ (riboflavin), 3.2 mg Vit B_6_ (pyridoxine), 0.011 mg Vit B_12_ (cyanocobalamin), 3.0 mg Vit K_2_ (menaquinone), 18 mg Vit B_5_ (pantothenic acid), 60 mg Vit B_3_ (nicotinic acid), 0.18 mg Vit B_7_ (biotin), 1.9 mg Vit B_9_ (folic acid),18 mg antioxidants (ethoxyquin).

3) Supplied per kg diet: 20 mg Fe, 16 mg Cu, 110 mg Zn, 120 mg Mn, 1.25 mg I, 0.9 mg Co, 0.3 mg Se.

**Table 2:** Ingredient and chemical composition of basal diet, as-fed basis (Phase II)

Items	Value				
Metabolizable energy (Kcal/kg)	3,200	3,150	3,150	3,100	3,100
Crude protein (%)	20.00	19.50	19.50	19.50	19.50
Enzyme (%)^1)^	0	0	0.05	0	0.05
Ingredients (%)					
Corn	50.68	55.32	55.27	56.47	56.42
Wheat	10.00	10.00	10.00	10.0	10.00
Soybean meal 45%	24.55	21.01	21.01	21.0	21.00
Soy-oil	5.00	2.99	2.99	2.02	2.02
Threonine 98.5%	0.06	0.08	0.08	0.07	0.07
Lysine 78%	0.26	0.32	0.32	0.32	0.32
Methionine 50%	0.51	0.50	0.50	0.49	0.49
Choline	0.05	0.05	0.05	0.05	0.05
Limestone	1.70	1.67	1.67	1.67	1.67
MDCP	1.33	1.13	1.13	1.12	1.12
Salt	0.20	0.20	0.20	0.20	0.20
Mineral premix^2)^	0.20	0.20	0.20	0.20	0.20
Vitamin premix^3)^	0.30	0.30	0.30	0.30	0.30
Corn gluten	5.16	6.23	6.23	6.09	6.09
Enzyme	-	-	0.05	-	0.05
Total	100.0	100.0	100.0	100.0	100.0
Chemical composition, calculated (%)					
Metabolizable energy	3,200	3,150	3,150	3,100	3,100
Crude protein	20.00	19.50	19.50	19.50	19.50
Calcium	0.90	0.85	0.85	0.85	0.85
Available phosphorus	0.40	0.35	0.35	0.35	0.35
SID Lysine	1.10	1.07	1.07	1.07	1.07
SID TSAA	0.84	0.81	0.81	0.81	0.81
Threonine	0.71	0.68	0.68	0.68	0.68
Tryptophan	0.20	0.18	0.18	0.18	0.18

MDCP, monodicalcium phosphate; SID, standardized ileal digestibiliy; TSAA, total sulfur amino acids; MNU, mannanase units; BAU, bacterial amylase units; FTU, phytase temperature units; PCU, protease catalytic units; XU, xylanase units

1) The multienzyme is a mix of mannanase, protease, amylase, phytase, and xylanase synthesised by controlled fermentation of *Bacillus amyloliquefaciens*, *Bacillus licheniformis*, *Trichoderma citrinoviride*, and *Pichia pastoris* respectively. The pure enzyme product provides 2,500,000 MNU/kg of β-mannanase, 800,000 BAU/kg of amylase, 1,000,000 FTU/kg of phytase, 1,000,000 PCU/kg of protease, 600,000 XU/kg of xylanase.

2) Supplied per kg diet: 10,000 IU Vit A (retinyl acetate), 4,500 IU Vit D_3_ (cholecalciferol), 65 mg Vit E (DL-α-tocopheryl acetate), 1.5 mg Vit B_1_ (thiamine), 12 mg Vit B_2_ (riboflavin), 3.2 mg Vit B_6_ (pyridoxine), 0.011 mg Vit B_12_ (cyanocobalamin), 3.0 mg Vit K_2_ (menaquinone), 18 mg Vit B_5_ (pantothenic acid), 60 mg Vit. B_3_ (nicotinic acid), 0.18 mg Vit B_7_ (biotin), 1.9 mg Vit B_9_ (folic acid), 18 mg antioxidants (ethoxyquin).

3) Supplied per kg diet: 20 mg Fe, 16 mg Cu, 110 mg Zn, 120 mg Mn, 1.25 mg I, 0.9 mg Co, 0.3 mg Se.

**Table 3:** Effects of nutrient density of diet and supplementation of exogenous enzyme blend on growth performance of broiler

Items	Value					SEM	p-value
ME (Kcal/kg, S/F)^1)^	3,100/3,200	3,060/3,150	3,060/3,150	3,010/3,100	3,010/3,100		
CP (%, S/F)^2)^	22.00/20.00	21.50/19.50	21.50/19.50	21.50/19.50	21.50/19.50		
Enzyme (%)^3)^	-	-	0.05	-	0.05		
Starter (d 1 to 2^1)^							
Body weight gain (g)	785^a^	740^bc^	791^a^	715^c^	779^ab^	6.31	<0.001
Feed intake (g)	1,115	1,152	1,129	1,144	1,127	11.66	0.903
FCR	1.42^b^	1.56^ab^	1.43^b^	1.60^a^	1.45^b^	0.02	0.002
Finisher (d 22 to 35)							
Body weight gain (g)	1,125^a^	1,078^ab^	1,128^a^	1,038^b^	1,119^a^	8.76	0.003
Feed intake (g)	2,082	2,088	2,062	2,054	2,071	6.39	0.402
FCR	1.85^b^	1.94^ab^	1.83^b^	1.98^a^	1.85^b^	0.02	0.006
Overall (d 1 to 35)							
Body weight gain (g)	1,910^a^	1,818^bc^	1,920^a^	1,752^c^	1,897^ab^	13.51	<0.001
Feed intake (g)	3,197	3,240	3,191	3,198	3,198	14.17	0.841
FCR	1.67^b^	1.78^a^	1.66^b^	1.83^a^	1.69^b^	0.01	<0.001

SEM, standard error of means; ME, metabolizable energy; S/F, starter/finisher; CP, crude proein; FCR, feed conversion ratio; MNU, mannanase units; BAU, bacterial amylase units; FTU, phytase temperature units; PCU, protease catalytic units; XU, xylanase units.

1) Dietary metabolizable energy level in starter and finisher stage.

2) Dietary crude protein level in starter and finisher stage.

3) The multienzyme is a mix of mannanase, protease, amylase, phytase, and xylanase synthesised by controlled fermentation of *Bacillus amyloliquefaciens*, *Bacillus licheniformis*, *Trichoderma citrinoviride*, and *Pichia pastoris* respectively. The pure enzyme product provides 2,500,000 MNU/kg of β-mannanase, 800,000 BAU/kg of amylase, 1,000,000 FTU/kg of phytase, 1,000,000 PCU/kg of protease, 600,000 XU/kg of xylanase.

a–c Values with different superscripts in the same row are different (p<0.05).

**Table 4:** Effects of nutrient density of diet and supplementation of exogenous enzyme blend on faecal and ileal digestibility of broiler (d 35)

Items	Value					SEM	p-value
ME (Kcal/kg, S/F)^1)^	3,100/3,200	3,060/3,150	3,060/3,150	3,010/3,100	3,010/3,100		
CP (%, S/F)^2)^	22.00/20.00	21.50/19.50	21.50/19.50	21.50/19.50	21.50/19.50		
Enzyme (%)^3)^	-	-	0.05	-	0.05		
Faeces (%)							
Dry matter	76.81	76.10	77.13	75.88	76.79	0.15	0.051
Gross energy	77.86	77.15	78.25	76.73	77.76	0.17	0.054
Crude protein	68.24^ab^	67.54^b^	68.82^a^	67.17^b^	68.25^ab^	0.19	0.049
Calcium	42.45	41.96	43.31	41.27	42.41	0.24	0.129
Phosphorus	40.34	39.68	41.41	38.98	40.40	0.26	0.061
Ileal (%)							
Dry matter	75.19	74.61	75.54	74.08	75.16	0.16	0.053
Gross energy	76.04^a^	75.66^ab^	76.47^a^	75.17^b^	75.88^ab^	0.14	0.046
Crude protein	66.67	66.22	67.29	65.65	66.64	0.18	0.076
Calcium	40.58	39.76	41.25	39.26	40.67	0.26	0.153
Phosphorus	38.73^ab^	38.24^b^	39.92^a^	37.40^b^	38.80^ab^	0.27	0.050

SEM, standard error of means; ME, metabolizable energy; S/F, starter/finisher; CP, crude proein; MNU, mannanase units; BAU, bacterial amylase units; FTU, phytase temperature units; PCU, protease catalytic units; XU, xylanase units.

1) Dietary metabolizable energy level in starter and finisher stage.

2) Dietary crude protein level in starter and finisher stage.

3) The multienzyme is a mix of mannanase, protease, amylase, phytase, and xylanase synthesised by controlled fermentation of *Bacillus amyloliquefaciens*, *Bacillus licheniformis*, *Trichoderma citrinoviride*, and *Pichia pastoris* respectively. The pure enzyme product provides 2,500,000 MNU/kg of β-mannanase, 800,000 BAU/kg of amylase, 1,000,000 FTU/kg of phytase, 1,000,000 PCU/kg of protease, 600,000 XU/kg of xylanase.

a–b Values with different superscripts in the same row are different (p<0.05).

**Table 5:** Effects of nutrient density of diet and supplementation of exogenous enzyme blend on blood metabolites in broiler

Items	Value					SEM	p-value
ME (Kcal/kg, S/F)^1)^	3,100/3,200	3,060/3,150	3,060/3,150	3,010/3,100	3,010/3,100		
CP (%, S/F)^2)^	22.00/20.00	21.50/19.50	21.50/19.50	21.50/19.50	21.50/19.50		
Enzyme (%)^3)^	-	-	0.05	-	0.05		
Glucose (μM/dL)							
21 d	128.88	123.68	127.57	129.03	124.72	1.60	0.297
35 d	135.97	131.39	133.13	136.94	133.27	1.53	0.163
Triglyceride (mg/dL)							
21 d	33.50	30.63	30.88	32.88	29.25	0.63	0.136
35 d	35.75	31.13	29.88	31.25	35.13	1.09	0.239
Cholesterol (mg/dL)							
21 d	123.50	128.75	129.00	123.25	128.50	1.24	0.415
35 d	124.75	127.25	122.00	121.63	128.88	1.27	0.327
Calcium (μM/dL)							
21 d	25.70	25.03	26.07	24.55	24.43	0.15	0.079
35 d	25.92	25.15	26.27	24.73	25.53	0.16	0.061
Phosphorus (μM/dL)							
21 d	2.85^a^	2.73^ab^	2.94^a^	2.61^b^	2.81^ab^	0.10	0.044
35 d	2.95^a^	2.81^ab^	3.01^a^	2.70^b^	2.89^ab^	0.10	0.049

SEM, standard error of means; ME, metabolizable energy; S/F, starter/finisher; CP, crude proein; MNU, mannanase units; BAU, bacterial amylase units; FTU, phytase temperature units; PCU, protease catalytic units; XU, xylanase units.

1) Dietary metabolizable energy level in starter and finisher stage.

2) Dietary crude protein level in starter and finisher stage.

3) The multienzyme is a mix of mannanase, protease, amylase, phytase, and xylanase synthesised by controlled fermentation of *Bacillus amyloliquefaciens*, *Bacillus licheniformis*, *Trichoderma citrinoviride*, and *Pichia pastoris*, respectively. The pure enzyme product provides 2,500,000 MNU/kg of β-mannanase, 800,000 BAU/kg of amylase, 1,000,000 FTU/kg of phytase, 1,000,000 PCU/kg of protease, 600,000 XU/kg of xylanase.

a–b Values with different superscripts in the same row are different (p<0.05).

**Table 6:** Effects of nutrient density of diet and supplementation of exogenous enzyme blend on intestinal microflora of broiler (d 35)

Items	Value					SEM	p-value
ME (Kcal/kg, S/F)^1)^	3,100/3,200	3,060/3,150	3,060/3,150	3,010/3,100	3,010/3,100		
CP (%, S/F)^2)^	22.00/20.00	21.50/19.50	21.50/19.50	21.50/19.50	21.50/19.50		
Enzyme (%)^3)^	-	-	0.05	-	0.05		
Total anaerobic bacteria (log10 cfu/g)							
Ileum	9.77^b^	10.07^a^	9.81^b^	10.13^a^	9.92^ab^	0.05	0.007
Cecum	9.86	10.19	9.83	10.16	9.94	0.06	0.168
*Lactobacillus* spp. (log10 cfu/g)							
Ileum	9.15	8.79	9.21	8.67	9.08	0.07	0.087
Caecum	9.49	9.07	9.55	8.94	9.45	0.08	0.095
Coliform bacteria (log10 cfu/g)							
Ileum	7.32	7.52	7.24	7.61	7.37	0.05	0.112
Cecum	7.41^bc^	7.64^ab^	7.34^c^	7.75^a^	7.46^bc^	0.05	0.007
*Clostridium* spp. (log10 cfu/g)							
Ileum	7.16	7.38	7.02	7.45	7.13	0.06	0.137
Caecum	7.41	7.73	7.35	7.60	7.44	0.06	0.187

SEM, standard error of means; ME, metabolizable energy; S/F, starter/finisher; CP, crude proein; MNU, mannanase units; BAU, bacterial amylase units; FTU, phytase temperature units; PCU, protease catalytic units; XU, xylanase units

1) Dietary metabolizable energy level in starter and finisher stage.

2) Dietary crude protein level in starter and finisher stage.

3) The multienzyme is a mix of mannanase, protease, amylase, phytase, and xylanase synthesised by controlled fermentation of *Bacillus amyloliquefaciens*, *Bacillus licheniformis*, *Trichoderma citrinoviride*, and *Pichia pastoris*, respectively. The pure enzyme product provides 2,500,000 MNU/kg of β-mannanase, 800,000 BAU/kg of amylase, 1,000,000 FTU/kg of phytase, 1,000,000 PCU/kg of protease, 600,000 XU/kg of xylanase.

a–c Values with different superscripts in the same row are different (p<0.05).

**Table 7:** Effects of nutrient density of diet and supplementation of exogenous enzyme blend on intestinal morphology of broiler (d 35)

Items	Value					SEM	p-value
ME (Kcal/kg, S/F)^1)^	3,100/3,200	3,060/3,150	3,060/3,150	3,010/3,100	3,010/3,100		
CP (%, S/F)^2)^	22.00/20.00	21.50/19.50	21.50/19.50	21.50/19.50	21.50/19.50		
Enzyme (%)^3)^	-	-	0.05	-	0.05		
Duodenum							
Villus height (μm)	1685.85	1652.09	1701.36	1624.07	1679.64	9.08	0.068
Crypt depth (μm)	146.49	158.76	144.90	167.00	156.00	2.95	0.128
VH/CD	11.71	10.52	11.94	9.88	10.91	0.27	0.119
Jejunum							
Villus height (μm)	837.28^a^	781.46^ab^	849.73^a^	742.43^b^	813.77^ab^	12.09	0.042
Crypt depth (μm)	108.92	114.10	103.56	112.43	106.12	1.49	0.180
VH/CD	7.79	6.92	8.30	6.69	7.78	0.21	0.112
Ileum							
Villus height (μm)	691.94	672.93	708.70	649.24	688.00	6.58	0.061
Crypt depth (μm)	100.23	102.43	96.50	101.19	96.54	0.98	0.242
VH/CD	6.94	6.61	7.38	6.45	7.18	0.13	0.155

SEM, standard error of means; VH, villus height; CD, crypt depth.

1) Dietary metabolizable energy level in starter and finisher stage.

2) Dietary crude protein level in starter and finisher stage.

3) The multienzyme is a mix of mannanase, protease, amylase, phytase, and xylanase synthesised by controlled fermentation of *Bacillus amyloliquefaciens*, *Bacillus licheniformis*, *Trichoderma citrinoviride*, and *Pichia pastoris*, respectively. The pure enzyme product provides 2,500,000 MNU/kg of β-mannanase, 800,000 BAU/kg of amylase, 1,000,000 FTU/kg of phytase, 1,000,000 PCU/kg of protease, 600,000 XU/kg of xylanase.

a–b Values with different superscripts in the same row are different (p<0.05).
